# Molecular detection and identification of *Trichobilharzia*: development of a LAMP, qPCR, and multiplex PCR toolkit

**DOI:** 10.1186/s13071-025-06822-y

**Published:** 2025-05-30

**Authors:** Jan Procházka, Zikmund Bartoníček, Roman Leontovyč, Petr Horák, Tomáš Macháček

**Affiliations:** Charles University, Prague, Czechia

**Keywords:** Cercarial dermatitis, Bird schistosomes, *Trichobilharzia*, LAMP, qPCR, Multiplex PCR, Monitoring, Detection

## Abstract

**Background:**

Cercarial dermatitis (CD), or swimmer’s itch, is a water-borne allergic skin reaction caused by the penetration of the larval stages of bird schistosomes (cercariae) into the skin. Members of the genus *Trichobilharzia* are the primary causative agents of CD worldwide. Due to the increasing number of cases, CD is regarded as a (re)emerging disease. Outbreaks in recreational waters can significantly impact public health and local economies. Environmental monitoring of *Trichobilharzia* is crucial for outbreak prediction and public health management. However, conventional methods, such as cercarial shedding and snail dissections, are labour-intensive and lack sensitivity. To overcome these limitations, we present a molecular toolkit that combines loop-mediated isothermal amplification (LAMP), quantitative polymerase chain reaction (qPCR), and multiplex PCR for rapid, sensitive, and accurate detection and identification of *Trichobilharzia* spp. from various biological samples.

**Methods:**

Tricho-LAMP and Tricho-qPCR were designed and optimised for *Trichobilharzia* DNA detection. A multiplex PCR assay was also developed and optimised to identify the three main species causing CD in Europe (*Trichobilharzia franki*, *T. szidati*, and *T. regenti*).

**Results:**

Tricho-LAMP specifically detected *T. regenti* and *T. franki* at 10^−3^ ng, and *T. szidati* at 10^−2^ ng per reaction with genomic DNA. Using gBlocks synthetic DNA, Tricho-LAMP achieved 100% amplification at 10,000 copies and 85% amplification at 1000 copies, with decreasing success at lower concentrations. Tricho-qPCR showed the highest sensitivity, detecting all species down to 10^−4^ ng per reaction and showing a limit of detection at 10 copies of synthetic DNA in the reaction. Multiplex PCR allowed reliable species differentiation via gel electrophoresis of the PCR products, but the assay had the lowest sensitivity.

**Conclusions:**

We provide a molecular toolkit consisting of LAMP, qPCR, and multiplex PCR. By exhibiting high sensitivity, Tricho-LAMP and Tricho-qPCR assays are potentially suitable for environmental DNA (eDNA)-based environmental monitoring of bird schistosomes, by both researchers and public health authorities. Multiplex PCR can be used for species determination without the need for further sequencing.

**Graphical Abstract:**

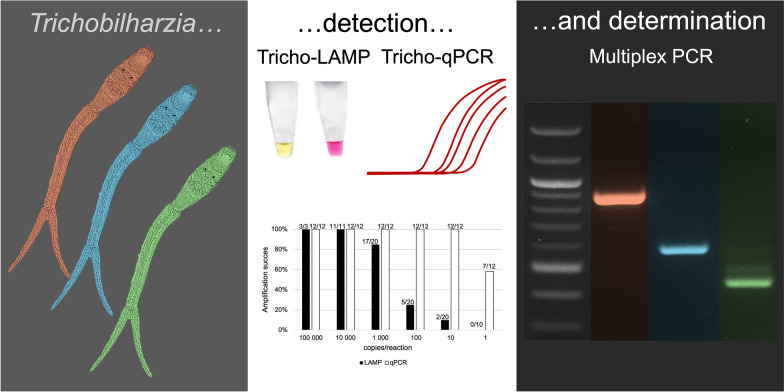

**Supplementary Information:**

The online version contains supplementary material available at 10.1186/s13071-025-06822-y.

## Background

Cercarial dermatitis (CD), commonly known as swimmer’s itch, is a hypersensitivity reaction caused by the penetration of cercariae, the free-swimming larval stages of trematodes, into the human skin [1, 2]. While mammalian species of schistosomes (such as *Schistosoma* spp.) can cause CD, especially in naive individuals [3], avian schistosomes (mainly *Trichobilharzia* spp.) are considered the major CD causative agents, with a global distribution [1]. These parasites primarily infect waterfowl as definitive hosts and utilise freshwater gastropods as intermediate hosts, completing their life cycle in aquatic ecosystems [4]. As accidental hosts, mammals restrict further migration and development, and most parasites are eliminated in the skin [5].

In humans, exposure to cercariae triggers allergic skin inflammation manifested by maculopapular/vesicular eruptions, oedema, and intense itching. In more sensitive individuals, even a generalised reaction may occur, including symptoms such as fever, cough, diarrhoea, and swelling of local lymph nodes [1, 2, 6]. Patients reporting repeated contact with cercariae and past pre-existing CD history show faster onset and higher severity of the symptoms [2]. This condition is particularly problematic in recreational water bodies, where outbreaks can significantly impact public health and local economies [7]. Moreover, due to the increasing number of cases in Europe [8–14] recently summarised by Bispo et al. [15] and elsewhere [16–18], CD is considered a (re)emerging disease [1, 14, 19].

Monitoring of *Trichobilharzia* in the environment is critical for predicting outbreaks and designing control strategies. Conventional detection methods, such as cercarial shedding assays and snail dissection [20], have remained standard in the field but have several limitations. These methods are labour-intensive and time-consuming, and often lack the sensitivity to detect low-level infections [20]. Consequently, molecular methods, including polymerase chain reaction (PCR) and quantitative PCR (qPCR), have gained attention for their ability to detect *Trichobilharzia* DNA with high sensitivity [21–23]. However, they require expensive equipment, trained personnel, and a laboratory environment, limiting their application in field settings or resource-limited areas. Recently, loop-mediated isothermal amplification (LAMP) has emerged as a straightforward, rapid, and cost-efficient method for pathogen detection, offering high sensitivity and specificity even with minimal input target DNA [24–27].

This study aims to provide a molecular toolkit for the detection and identification of *Trichobilharzia* spp., the primary CD causative agents, from various biological samples, potentially including cercariae or filtered environmental DNA (eDNA). First, we established a novel LAMP assay for the detection of *Trichobilharzia* DNA theoretically without the need for specialised laboratory equipment. We also developed a *Trichobilharzia*-specific qPCR assay, which allows users to quantify and detect *Trichobilharzia* DNA even in very low concentrations, making it possibly suitable for eDNA-based settings. Lastly, we present an end-point multiplex PCR assay designed to enable rapid identification of the three most common *Trichobilharzia* species in Europe—*Trichobilharzia franki*, *T. szidati*, and *T. regenti*—via gel electrophoresis without the need for sequencing. Identifying specific species enables the design of more precise treatment and management strategies due to their varying intermediate host preferences. Altogether, these assays equip users, ranging from field researchers to public health authorities, with a molecular toolkit that can be potentially used in a variety of scenarios, including environmental monitoring of CD risk or species identification.

## Methods

### Organisms used in the study

Cercariae of routinely maintained laboratory strains of *T. szidati* and *T. regenti* [28, 29] were used as reference genomic DNA (gDNA), along with field-collected (Czechia) cercariae of *T. franki*. For specificity testing, gDNA was also extracted from laboratory-maintained *Schistosoma mansoni* (Puerto Rico strain, [30]) and field-collected (Czechia) cercariae of *Bilharziella polonica*, *Allobilharzia visceralis*, *Australapatemon burti*, *Echinostoma revolutum*, *Plagiorchis maculosus*, and *Hypoderaeum conoideum* cercariae. All DNA samples were extracted with the Exgene™ Tissue SV Plus mini kit (GeneAll, Korea) following the manufacturer’s protocol, and the DNA concentration was normalised to 0.5 ng/μl. The identities of all the samples were confirmed via Sanger sequencing of the internal transcribed spacer (*ITS*) region [31] and subsequent nucleotide Basic Local Alignment Search Tool (BLASTn) analysis against the GenBank database (http://www.blast.ncbi.nlm.nih.gov/).

### Synthetic DNA templates for sensitivity tests: gBlocks

**Fig. 1:**
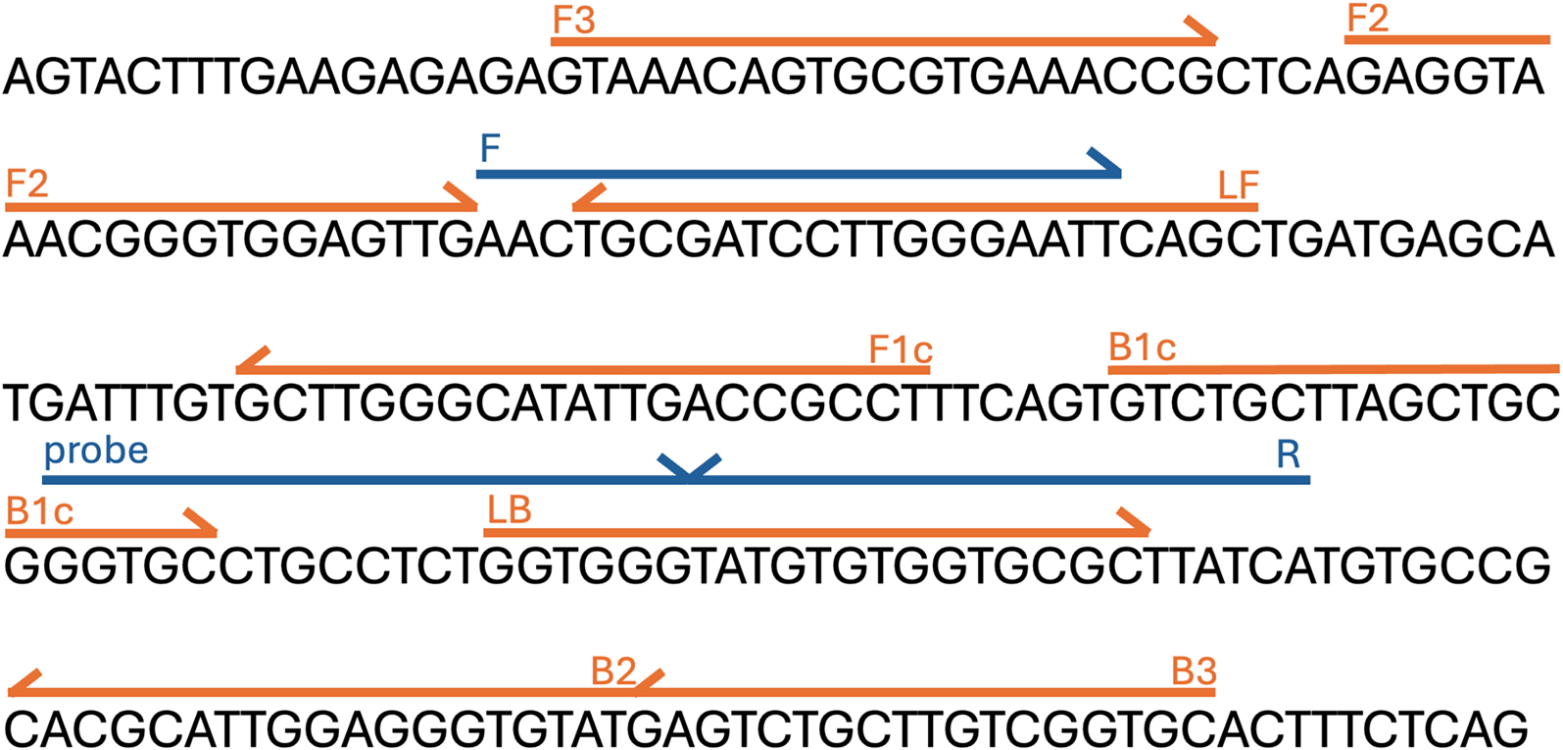
Partial sequence of the *28S* region used for Tricho-LAMP and Tricho-qPCR assay design (gBlocks sequence 237 bp, black capital letters) showing the position of LAMP primers (orange) and qPCR primers and probe (blue) on the sequence

Synthetic double-stranded DNA (gBlocks) was synthesised (IDT, UK) to serve as a standard with a known DNA copy number. The 237-base-pair (bp)-long sequence (Fig. 1), which is based on *T. franki* (KJ775867), was selected to represent the fraction of the *Trichobilharzia* spp. *28S* region targeted by the qPCR and LAMP assays, with 10 or more flanking bases on each end. Before use, gBlocks were diluted to multiple aliquots with 10^8^ copies/μl, which were stored at −20 °C until use. When gBlocks were used, serial dilutions of 1–10^5^ copies/ml were prepared, stored at 4 °C, and used within 1 week.

### *Trichobilharzia* LAMP and qPCR primer design

The *28S* ribosomal RNA (rRNA) gene was selected for designing *Trichobilharzia* genus-specific LAMP (Tricho-LAMP) and qPCR (Tricho-qPCR) assays. The GenBank sequences of *T. regenti* (HM439491), *T. szidati* (FJ174476), and *T. franki* (KJ775867) were aligned in Geneious Prime 2024.0.5 via the Clustal Omega algorithm, and the most conserved regions were selected for primer design. The LAMP primer design was performed via the online New England Biolabs (NEB) LAMP Primer Design Tool, version 1.4.2 (https://lamp.neb.com/). All LAMP primers were synthesised by Merck (custom DNA oligos). The same alignment of *Trichobilharzia* sequences used for LAMP (see above) was used for the design of the qPCR primers and probe. The candidate primers and TaqMan probes were designed using the Primer3 plugin of Geneious Prime 2024.0.5, with the amplicon length set between 110 and 150 bp and optimal melting temperatures (T_m_) set at 55–65 °C for primers and 64–74 °C for the probe. Designed primers and probes were inspected in Geneious Prime 2024.0.5 for mismatches against the *Trichobilharzia* reference sequences. The specificity to the intended targets was also confirmed via a BLASTn search against the GenBank database of the selected primer and probe combinations, confirming that no other genera should be amplified. The synthesised probe (IDT, UK) was labelled with a fluorescein amidite (FAM) fluorophore, and internal Iowa Black FQ (IBFQ) and ZEN quenchers were used to reduce background fluorescence. The sequences of the primers and probe are shown in Fig. 1 and Table 1.

**Table 1:** Primer sets for LAMP, qPCR, and multiplex end-point PCR

Assay	Primer name	Nucleotide sequence (5′ to 3′)
Tricho-LAMP	FIP (F1c + F2)	AGGCGGTCAATATGCCCAAGC-GAGGTAAACGGGTGGAGTTG
	BIP (B1c + B2)	GTCTGCTTAGCTGCGGGTGC-ATACACCCTCCAATGCGTG
	LF	GCTGAATTCCCAAGGATCGCA
	LB	GGTGGGTATGTGTGGTGCGC
	F3	GTAAACAGTGCGTGAAACCG
	B3	GCACCGACAAGCAGACTC
Tricho-qPCR	T28SF	AACTGCGATCCTTGGGAATT
	T28SR	TGATAAGCGCACCACACATA
	T28SPr	/56-FAM/CCCACCAGA/ZEN/GGCAGGCACC/3IABkFQ/
Multiplex PCR	TFdeg	ATGGGGGTDGGKGTTGATT
	TRegR	ACACGACGTGGCAATCCAT
	TFraR	GCTCAACCCCACGCAAATAC
	TSziR	ACGTAAACAAATACGCCCAAACA

### Multiplex PCR primer design

The cytochrome c oxidase subunit 1 gene (*cox1*) was selected as the target for the European *Trichobilharzia* determination assay due to its higher interspecific variability than the *28S* region. GenBank sequences of *T. regenti* (PP232102.1), *T. franki* (OP347092.1), and *T. szidati* (MT708496.1) were aligned via MUSCLE in Geneious Prime. A degenerate forward primer was designed to amplify all three target species, with species-specific reverse primers targeting loci that had at least two mismatches compared with the other two species. To enable multiplexing in end-point PCR and identification via agarose gel electrophoresis, each species-specific amplicon was designed to have a distinct length: 100–200 bp for *T. szidati*, 500–600 bp for *T. franki*, and 700–900 bp for *T. regenti*. Primer inclusion criteria were the T_m_ of 55–65 °C and at least three mismatches against non-target species. Specificity was then checked in silico via BLAST against the GenBank database.

### LAMP reactions

Each LAMP reaction was performed with WarmStart^®^ Colorimetric LAMP 2× Master Mix with UDG (NEB, UK) in a laboratory thermoblock (ThermoMixer C, Eppendorf, Germany). The reaction mixture contained 7.5 μl of Master Mix, 1.5 μl of 10× Primer Mix (16 μM FIP/BIP, 2 μM F3/B3, and 4 μM LF/LB), 1 μl of DNA target (0.5 ng/μl), 1.125 μl dimethyl sulfoxide (DMSO) and water to a final volume of 15 μl. Optimised conditions were 67 °C and 40 min. A temperature gradient (65–68 °C) testing was performed to identify the optimal conditions for 40 min of amplification. After amplification temperature optimisation, different concentrations of DMSO (5%, 7.5%) were tested to reduce false positives. The results were determined by reaction colour observation, with pink indicating a negative result and yellow indicating a positive result. All experiments included no template and positive controls consisting of PCR H_2_O and DNA standards, respectively. To confirm the presence of the DNA amplicons, the whole reaction volume was run in gel electrophoresis (2% agarose gel, 60 min, 90 V) and visualised with SYBR Green I nucleic acid gel stain to identify the typical ladder-like pattern of the amplicons.

### qPCR reactions

Each qPCR reaction was performed in a total volume of 10 μl and consisted of 5 μl NEB Luna^®^ Universal Probe qPCR Master Mix (NEB, UK), 2 μl of DNA template, 0.5 μl of each T28SF and T28SR primer (10 μM), 0.2 μl of the T28SPr probe (10 μM), and 1.8 μl PCR H_2_O. Cycling and fluorescence analysis were performed in a LightCycler^®^ 480 II Instrument (Roche, USA) following an adapted MasterMix manufacturer’s protocol: 1-min initial denaturation at 95 °C, followed by 45 cycles of 95 °C for 15 s and combined annealing and extension at 60 °C for 45 s, with fluorescence reading at the end of each cycle. The reaction was considered positive if the CP (crossing point, equivalent to the cycle threshold) value was lower than 37. All experiments included no template and positive controls consisting of PCR H_2_O and DNA standards, respectively.

### Multiplex PCR reactions

Each PCR reaction was performed in a total volume of 20 μl. The reaction mixture was as follows: 10 μl of EmeraldAmp MAX PCR Master Mix (Takara Bio, Japan), 1 μl of TFdeg forward primer (10 μM), 1 μl of TRegR reverse primer (10 μM), 1 μl of TFraR reverse primer (10 μM), 1 μl of TSziR reverse primer (10 μM), 3 μl of PCR H_2_O, 1 μl of DMSO, and 2 μl of template DNA. PCR cycling was done in a C1000 Touch Thermal Cycler (Bio-Rad, USA). Optimised cycling conditions were as follows: 2 min of initial denaturation at 95 °C; 35 cycles of 30 s at 95 °C, 15 s at 57 °C, and 60 s at 72 °C; and a final extension of 9 min at 72 °C. To identify the optimal reaction mixture and cycling conditions to avoid non-specific amplification, gradient PCRs (with annealing temperatures 55–63 °C) and various concentrations of DMSO (0–10% of the total reaction volume) were performed. No template controls and positive controls were used during each experiment. Amplicons (20 μl) were visualised via gel electrophoresis (2% agarose gel stained with Green I nucleic acid gel stain, 75 min, 90 V).

### Specificity and sensitivity testing

For the specificity testing of all the assays, gDNA from different *Trichobilharzia* and non-*Trichobilharzia* trematodes was used (see “Organisms used in the study” above). After normalising the DNA concentration (0.5 ng/μl), 1 ng of the template was used in each assay (PCR, qPCR, and LAMP).

Two types of templates were used for sensitivity testing: (i) dilution series of gDNA from 1 ng to 10^−4^ ng per reaction (LAMP, qPCR, PCR) or (ii) gBlocks standards at concentrations of 10^5^ to 1 copy (LAMP, qPCR). Using these templates, we established the limit of detection (LoD_95_, the lowest concentration at which the assay amplifies in 95% or more cases). The experiments with gDNA were performed in triplicate for all species and methods. To establish the LoD using gBlocks for qPCR, 12 replicates were performed for each concentration, whilst in the case of LAMP, 3–20 replicates were run in concentrations around which the LoD was expected.

## Results

### Tricho-LAMP

**Fig. 2:**
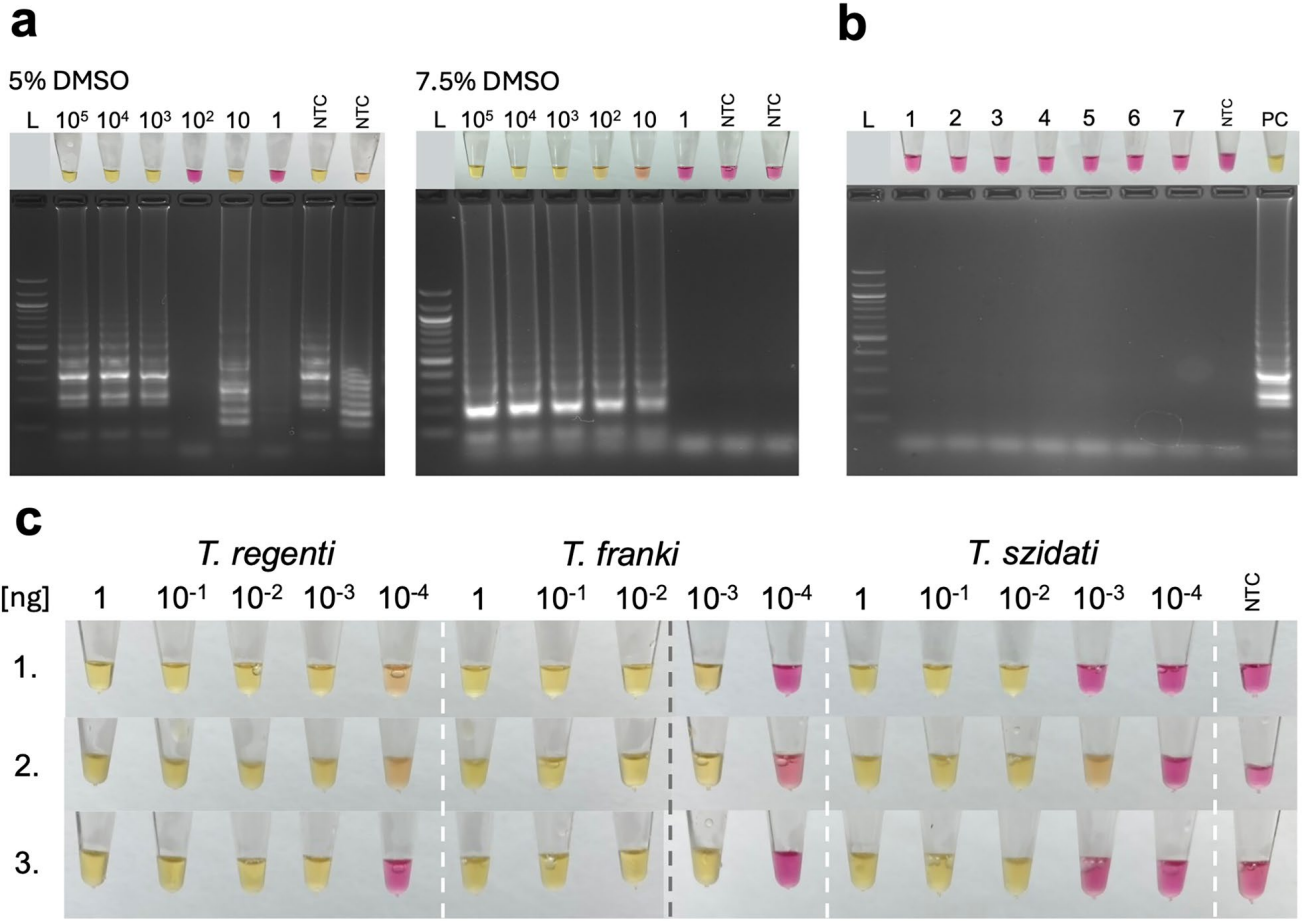
Tricho-LAMP assay results: yellow indicates a positive result and pink a negative result. **a** Reaction optimisation: Different concentrations of DMSO used on dilution series of gBlocks from 10^5^ to 1 copy per reaction. L: NEB 100 bp ladder; NTC: no template control. Using 5% DMSO, non-specific patterns occur on gel electrophoresis; therefore, 7.5% DMSO was selected as optimum. **b** Specificity testing on non-target trematodes: 1: *Allobilharzia visceralis*, 2: *Bilharziella polonica*, 3: *Australapatemon burti*, 4: *Echinostoma revolutum*, 5: *Plagiorchis maculosus*, 6: *Hypoderaeum conoideum*, 7: *Schistosoma mansoni*. L: 100-bp DNA ladder, PC: positive control (1 ng *Trichobilharzia szidati* gDNA), NTC: no template control. **c** Specificity and sensitivity testing on dilution series (1–10^−4^ ng) of gDNA of three species (*Trichobilharzia regenti*, *T. franki*, and *T. szidati*) separated by white dashed lines. Each row (1–3) corresponds to one replicate; the grey dashed line indicates stitching between individual strips of eight tubes. *NTC* no template control

Optimisation of the reaction conditions: First, a temperature gradient from 65–68 °C was used. As shown in Additional File 1, Figure S1, the lower temperatures (65–66 °C) led to strong false-positive results. At the higher temperatures (67–68 °C), the results were negative by reaction colour, but weak positivity was visible via gel electrophoresis. Different concentrations of DMSO were tested to reduce the false positives, and the optimum was identified as 7.5% DMSO (1.125 μl/reaction) (Fig. 2a). Hence, further reactions were run at 67 °C with the addition of 7.5% DMSO.

Specificity testing: *Trichobilharzia* LAMP was evaluated by testing DNA amplification extracted from seven non-target trematodes and three *Trichobilharzia* species. Only amplification of *Trichobilharzia* species was detected by colour change (Fig. 2b, c) and gel electrophoresis (Additional file 2, Fig. S2).

Sensitivity testing: The gDNA dilution series demonstrated varying limits among the three *Trichobilharzia* species (Fig. 2c). The highest sensitivity was observed for *T. regenti*, which was detected at concentrations as low as 10^−4^ ng in two out of three replicates (2/3). For *T. franki*, the detection limit was 10^−3^ ng across all three replicates; however, 10^−4^ ng was faintly positive by colour with a typical amplicon pattern on gel electrophoresis in one replicate (Additional file 2, Fig. S2). The lowest sensitivity was observed for *T. szidati*, with a reliable detection limit of 10^−2^ ng and one positive replicate at 10^−3^ ng per reaction.

**Fig. 3:**
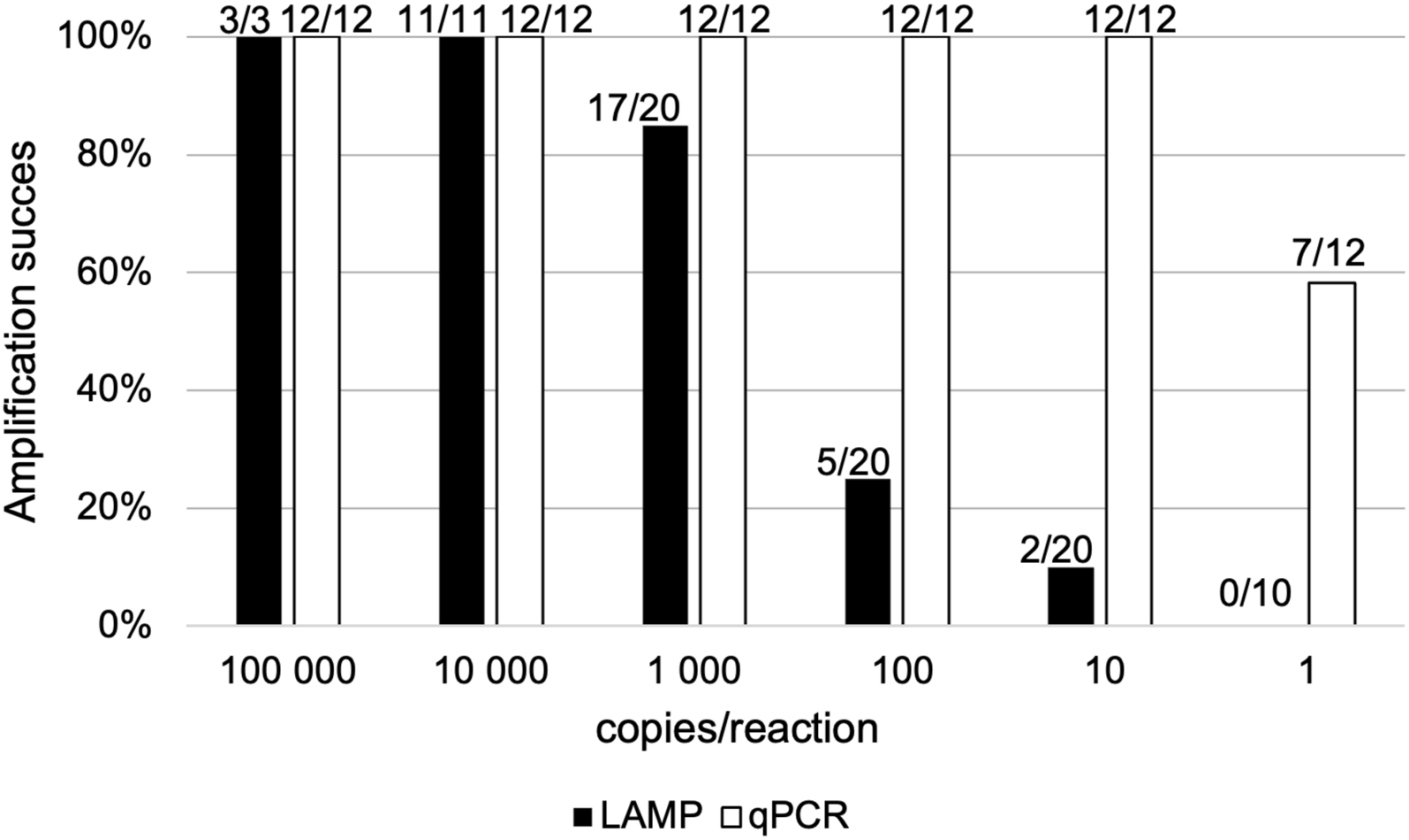
Sensitivity and amplification success of LAMP and qPCR with gBlocks DNA standards. Black boxes indicate the percentage amplification success of Tricho-LAMP with various DNA standard dilutions, while grey boxes show the percentage amplification success of Tricho-qPCR. Values above individual boxes represent the ratio between positive and all reactions

The LoD95 of the Tricho-LAMP assay was also tested via a dilution series of gBlocks synthetic standards. The assay achieved 100% amplification success (3/3 positive reactions) at 100,000 copies per reaction and (11/11 positive reactions) at 10,000 copies per reaction. The amplification success rate decreased at lower template concentrations, yielding 17/20 positive reactions at 1000 copies, 5/20 at 100 copies per reaction, 2/20 at 10 copies per reaction, and no amplification at one copy per reaction. A higher number of replicates was performed in the concentrations around which the LoD was expected. The results are summarised (and compared to qPCR, see below) in Fig. 3.

### Tricho-qPCR

Optimisation of the reaction conditions: First, the primer concentrations were optimised. The manufacturer’s recommended concentration (0.4 μM) was increased to 0.5 μM, as the original concentration resulted in suboptimal assay efficiency (< 90%). With the adjusted mixture, the average reaction efficiency was 93% (90–96%), and the average error was 3.6%.

Specificity testing: The Tricho-qPCR assay amplifies all species of *Trichobilharzia* tested in this study: *T. regenti*, *T. szidati*, and *T. franki*. The assay amplified none of the non-target trematode DNA tested here.

**Fig. 4:**
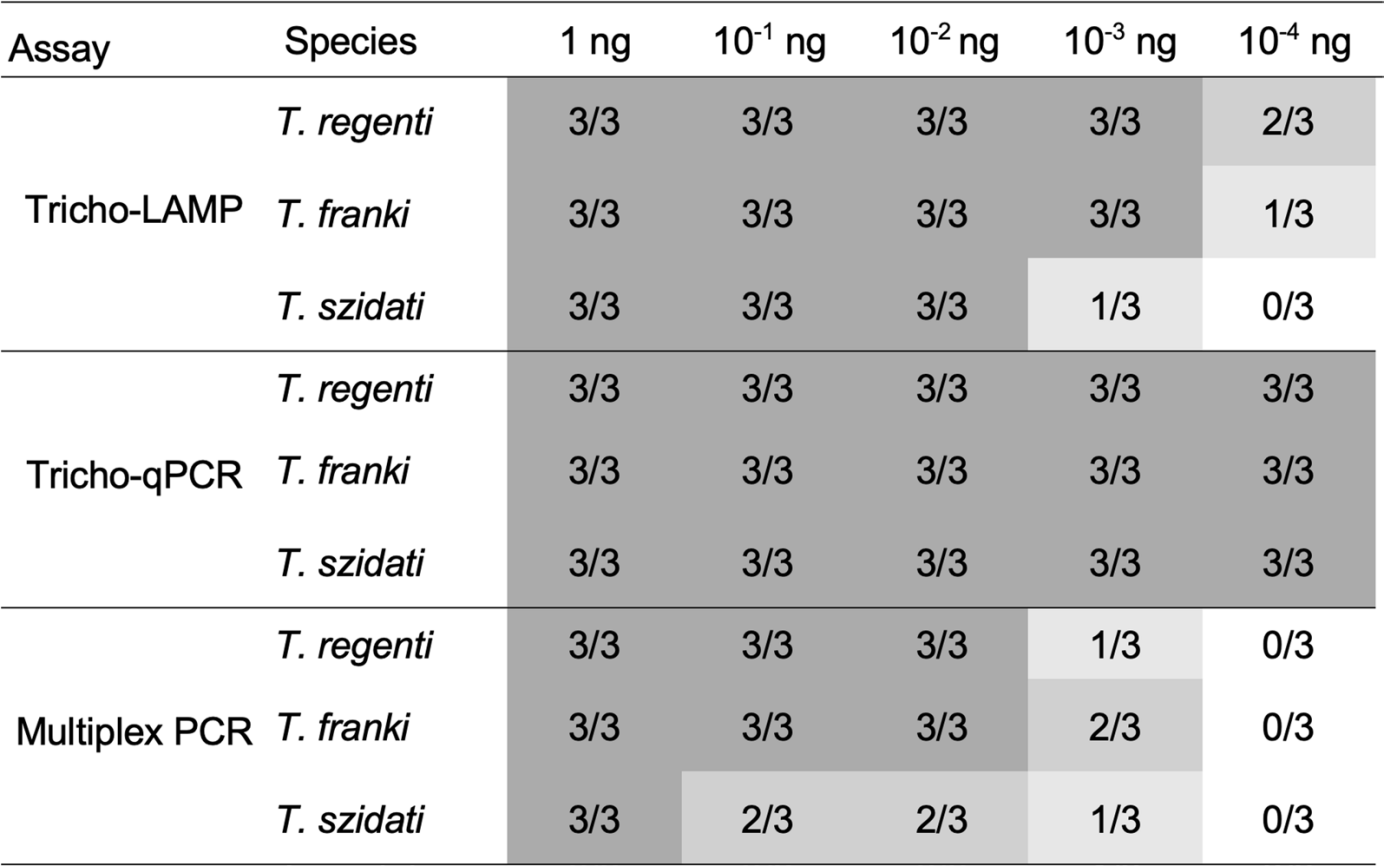
Sensitivity comparison of all amplification methods used on dilution series of gDNA (1 ng, 10^−1^ ng, 10^−2^ ng, 10^−3^ ng, and 10^−4^ ng) of individual species. The number in boxes represents amplification success for all reactions. The grey shading corresponds to the positivity ratio of all reactions. Tricho-qPCR was successful in all reactions; the results of Tricho-LAMP and multiplex PCR varied between species

Sensitivity testing: The assay is highly sensitive, successfully amplifying the DNA of all three *Trichobilharzia* species tested at concentrations as low as 10^−4^ ng/reaction. The results are summarised in Fig. 4. When sensitivity was tested using the gBlocks synthetic standards, the Tricho-qPCR amplified all tested concentrations with 10 or more copies of the template DNA in all the replicates, and a single copy of *Trichobilharzia* DNA was detected in seven of the 12 reactions.

### Multiplex end-point PCR

**Fig. 5:**
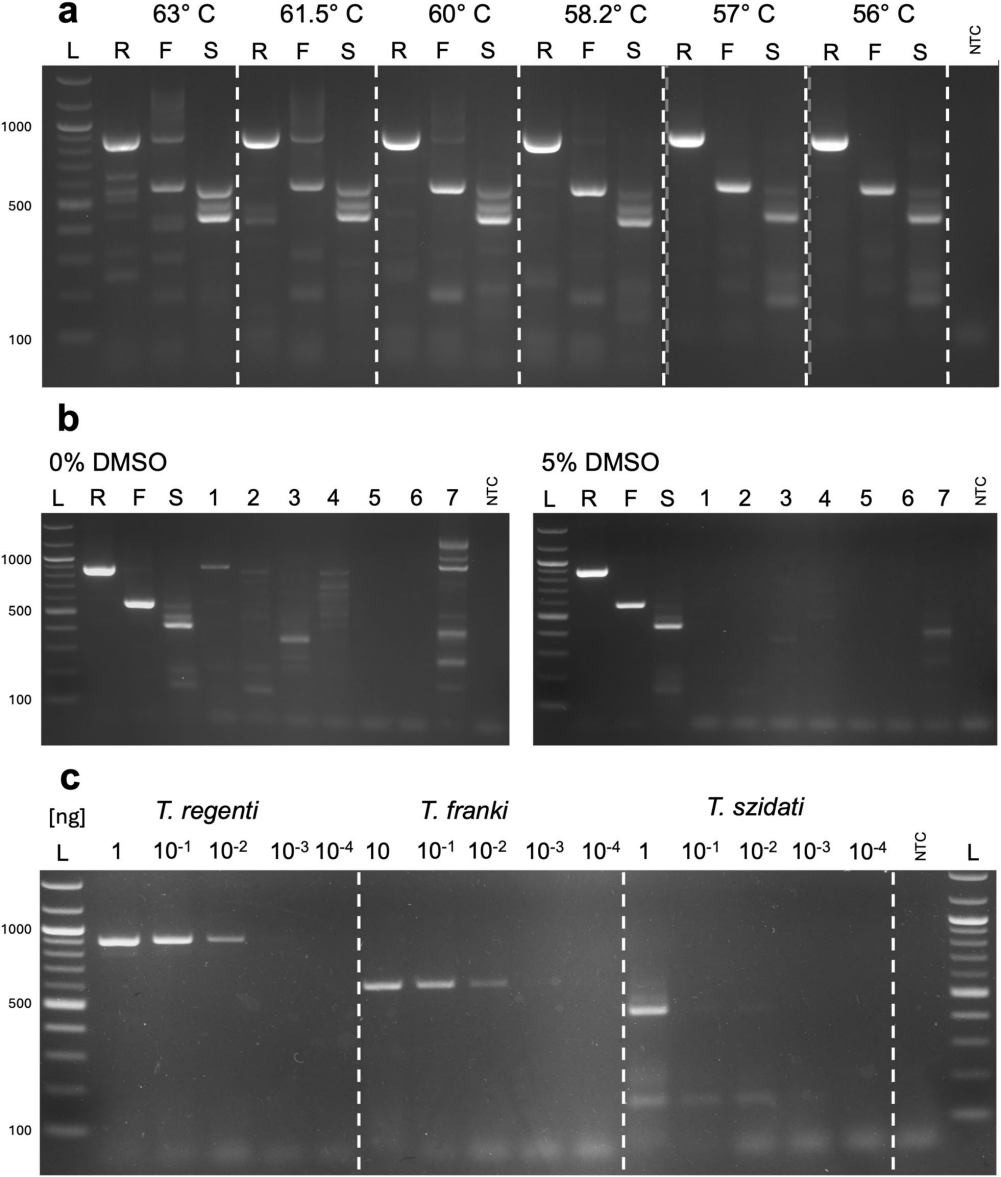
Optimisation and testing of the multiplex Tricho-PCR. **a** A composite gel image showing the results of gradient PCRs in identifying optimal annealing temperatures for *Trichobilharzia regenti* (R), *T. franki* (F), and *T. szidati* (S). Tested temperatures are shown above individual gel sections separated by white dashed lines. Grey dashed lines show stitching between individual gels (full gels are shown in the supplementary material). L: GeneRuler DNA Ladder Mix (Thermo Scientific), NTC: no template control. **b** Gels showing specificity tests with 1 ng of DNA template at different DMSO concentrations (0% and 5%). L: 100-bp DNA ladder (NEB), R: *T. regenti*, F: *T. franki*, S: *T. szidati*. 1: *Allobilharzia visceralis*, 2: *Bilharziella polonica*, 3: *Australapatemon burti*, 4: *Echinostoma revolutum*, 5: *Plagiorchis maculosus*, 6: *Hypoderaeum conoideum*, 7: *Schistosoma mansoni*, NTC: no template control. The characteristic banding patterns for *T. regenti* (~890 bp), *T. franki* (~580 bp), and *T. szidati* (145 + ~400 bp) remain unchanged at both concentrations. 5% DMSO markedly reduces non-specific amplification, and only *S. mansoni* produces amplicons (~400 bp) similar to *T*. *szidati*. **c** Sensitivity tests of multiplex Tricho-PCR for individual species. The amount of added DNA (1 ng, 10^−1^ ng, 10^−2^ ng, 10^−3^ ng, and 10^−4^ ng) is shown above individual lanes, and species are indicated by a white dashed line. Characteristic banding for individual species can be observed, with 400-bp amplicon in *T. szidati* fading at concentrations < 1 ng, whilst a 145-bp amplicon is observable up to 10^−3^ ng. L: 100-bp DNA ladder (NEB), *NTC* no template control. Only one of three replicates is shown here

The multiplex assay producing distinct banding patterns for different *Trichobilharzia* species was successfully designed. Gradient PCR was used to identify the optimal annealing temperature; 57 °C was selected because amplicons of the expected sizes were most distinct, and the fewest unintended amplicons were observed at this temperature (Fig. 5a). However, initial specificity tests resulted in non-specific amplification. Therefore, different concentrations of DMSO were tested, and 5% DMSO was identified as the optimal concentration (Fig. 5b). This concentration removed the majority of non-specific amplicons, except for a weak amplification of *S. mansoni*, which is currently not known to be co-endemic with *Trichobilharzia* in Europe [32]. When higher concentrations of DMSO were tested, the amplification of *Trichobilharzia* spp. was impaired (see Additional file 3: Fig. S3 for detailed results). Under optimised conditions (57 °C annealing temperature, 5% DMSO), the multiplex assay produced distinct banding patterns for each tested species: *T. regenti* produced an 893-bp-long amplicon, *T. franki* had a characteristic 580-bp-long amplicon, and *T. szidati* produced a combination of the anticipated 145-bp-long amplicon and an approximately 400-bp-long amplicon, which was not expected from in silico tests and was only present in very high DNA concentrations (Fig. 5c).

The sensitivity of the assay was tested in triplicate, and all primer pairs reliably amplified 1 ng of target gDNA. The multiplex assay reliably amplified *T. regenti* DNA at concentrations as low as 10^−2^ ng per reaction, with one replicate amplifying at 10^−3^ ng per reaction and no amplification at lower concentrations. For *T. franki*, all replicates up to 10^−2^ ng per reaction were amplified, with a faint band visible in 2/3 reactions with 10^−3^ ng of *T. franki* gDNA. *Trichobilharzia szidati* was amplified in all replicates only when 1 ng of template DNA was added. At lower concentrations, two of three replicates amplified *T. szidati* DNA up to 10^−2^ ng, and one replicate amplified 10^−3^ ng of *T. szidati* DNA. None of the tested primer pairs detected 10^−4^ ng of target DNA per reaction (Fig. 5c). The results are summarised in Fig. 4.

### Overall performance comparison

All optimised assays are specific for the genus *Trichobilharzia* (LAMP, qPCR) or some of the three selected species (PCR), although the sensitivity varies among the different methods. Figure 4 shows a comparison of the sensitivity of individual assays tested on the gDNA of three *Trichobilharzia* species.

The most sensitive assay is Tricho-qPCR, which detected all tested concentrations (1–10^−4^ ng) for all three species without differences in performance. Tricho-LAMP also demonstrated high sensitivity but showed a slight decrease in detection at the lowest concentrations, particularly for *T. szidati*. Multiplex PCR was the least sensitive, as detection decreased notably at lower DNA concentrations, especially for *T. szidati*.

To further assess sensitivity, LAMP and qPCR assays were tested using synthetic gBlocks (Fig. 3). These results reinforce the trends observed with gDNA. qPCR consistently detected even the lowest DNA concentrations, amplifying down to one copy in some cases. LAMP performed well at high template concentrations but showed declining success below 1000 copies, failing at the single-copy level.

## Discussion

Monitoring of *Trichobilharzia* spp., the predominant causative agent of CD, is crucial for predicting outbreaks, assessing exposure risk, and designing control strategies. In this study, we developed a molecular toolkit consisting of LAMP, qPCR, and multiplex PCR to detect and identify *Trichobilharzia* spp. These methods offer advantages to overcome the limitations of conventional snail-based detection methods by providing higher sensitivity and eliminating the need for the collection of live intermediate hosts. Although molecular approaches for *Trichobilharzia* DNA detection have been explored previously [22, 23, 33–35], our study advances these methods by introducing LAMP with the potential for rapid detection, optimising qPCR for high-sensitivity quantification, and developing a multiplex PCR assay for species-level identification without sequencing. The combination of these approaches enhances the flexibility and applicability of *Trichobilharzia* detection in various scenarios.

Our toolkit specifically targets *Trichobilharzia* spp., as it is the cause of most CD cases in Europe [1]. The ability to cause CD has also been discussed in other avian schistosome genera, but the epidemiological and experimental data supporting these claims are not always convincing [6]. In silico specificity tests, performed by BLASTing our sequences against the National Center for Biotechnology Information (NCBI) database, indicate that the selected target region is conserved across multiple *Trichobilharzia* species and is not restricted to the three species tested in vitro. This broad specificity is important, as CD can be caused by several different *Trichobilharzia* species worldwide, including *T. szidati*, *T. franki*, *T. regenti*, *T. querquedulae*, *T. stagnicolae*, and *T. physellae* [6, 15, 16]. Further empirical validation with DNA from additional *Trichobilharzia* species is needed to confirm the full applicability of our toolkit for genus-specific detection.

Among the tested methods, the Tricho-LAMP assay was developed as a rapid and sensitive tool for potential detection of *Trichobilharzia* spp. in eDNA samples. However, further validation using eDNA-based samples is necessary, as in this study we tested the assays only with total genomic extracts and synthetic gBlocks DNA. While LAMP has been successfully applied for detecting human schistosomes [26, 36], a LAMP assay specifically targeting *Trichobilharzia* DNA has not been established until now. We developed the first Tricho-LAMP assay for genus-specific detection, filling a critical gap in molecular monitoring tools for *Trichobilharzia* spp. The isothermal colourimetric format was selected to enable simple visual interpretation, making it particularly suitable for field applications where qPCR-based methods may be impractical. The colourimetric LAMP assay has the highest potential for use in the field, but suitable methods are still required, such as field-friendly DNA isolation, freeze-dried reaction mixes, and a thermal block with a battery. However, despite the advantages of these assays, the results do not reach the extreme sensitivities (down to 10^−6^ ng) reported elsewhere [37] and vary among the tested species (10^−3^ ng for *T. franki* and *T. regenti*, and 10^−2^ ng for *T. szidati*). Nevertheless, similar sensitivity (3.49 × 10^−4^ ng/μl) was achieved in the LAMP assay for the successful detection of the aquatic snail *Galba truncatula* in eDNA samples [38]. In the context of sensitivity tested on synthetic DNA, the Tricho-LAMP assay was able to amplify up to 10 copies, but the LoD_95_ was only 10,000 copies per reaction. Based on the number of *18S* gene (part of the same ribosomal DNA [rDNA] repeat unit as *28S* that we used) copies in *T. stagnicolae*—55,851 copies per cercaria [21]—we estimate the capability of our LAMP assay to amplify DNA amounts smaller than that in a single whole cercaria. The slightly lower sensitivity of our assay can be attributed to the addition of DMSO to suppress false positives, which can reduce the sensitivity of the reaction [39]. Nevertheless, LAMP remains a highly effective detection tool, potentially in large-scale field screening surveys where speed and accessibility are critical.

For scenarios where higher sensitivity and quantification of DNA copy number are needed, we developed the Tricho-qPCR assay. This assay has the highest sensitivity of all of our tested methods, with the capability for amplification up to one copy and a LoD95 of 10 copies, reaching similar sensitivity as in previously reported qPCR assays [21, 34]. Compared with other studies, our assay specifically amplifies different samples belonging to the genus *Trichobilharzia*, whereas the existing assays are species-specific [34] or detect avian schistosomes more broadly, including other genera [21]. As discussed above, *Trichobilharzia* genus specificity is most important for European control strategies. Compared with Tricho-LAMP, Tricho-qPCR offers superior sensitivity but requires a qPCR thermocycler, making it less suitable for field-based monitoring than LAMP. Despite this limitation, qPCR remains the gold standard for eDNA studies due to its robustness and quantitative capabilities [40].

While the assays detailed above (LAMP, qPCR) provide the user with tools to detect *Trichobilharzia* at the genus level, our multiplex PCR assay is a tool for species-level determination. As morphological identification of *Trichobilharzia* cercariae is extremely difficult and practically impossible [41], sequence analysis may help to solve this problem—for example, sequencing of conserved regions for family- or higher-level taxa, such as *ITS* or *28S* [31, 42]. This approach is time-consuming and requires multiple steps and sequencing. To enable rapid identification of predominant *Trichobilharzia* species in Europe (*T. szidati*, *T. franki*, and *T. regenti*) without the need for sequencing, we developed a multiplex PCR, where species identification is possible based solely on the size of the PCR amplicons. This approach was previously used for xenomonitoring in *Schistosoma*-infected snails or identification of *Schistosoma* hybrids [43–46], but has not been used for species identification within the genus *Trichobilharzia*. This rapid approach enhances our toolkit, complementing LAMP and qPCR for comprehensive monitoring and significantly shortening the time required for species determination.

Overall, the assays in our molecular toolkit provide a complex approach to *Trichobilharzia* detection. LAMP enables rapid parasite presence/absence screening, qPCR allows for sensitive DNA quantification, and multiplex PCR facilitates species identification without the need for sequencing. Combining LAMP and PCR can be particularly useful in a rapid two-step setup for detecting and determining the genus and species, respectively.

## Conclusions

We developed a molecular toolkit for *Trichobilharzia* detection consisting of (i) LAMP and qPCR for sensitive and specific genus detection, and (ii) multiplex PCR for species identification (*T. franki*, *T. szidati*, and *T. regenti*). Together, these methods could enhance control strategies for CD, contributing to European ecological/biodiversity studies and public health management.

## Supplementary Information

Additional file 1: Fig. S1. Temperature gradient used to select the optimal temperature for reduction of false-positives. Tested temperatures are shown above individual sections separated by white dashed lines. L: 100-bp DNA ladder, PC: positive control (1 ng *Trichobilharzia* gDNA), NTC: no template control in duplicate.

Additional file 2: Fig. S2. Specificity and sensitivity testing on dilution series (1–10^−4^ ng) of gDNA of three species (*Trichobilharzia regenti*, *T. franki*, and *T. szidati*). Each row corresponds to one replicate. NTC: no template control. Grey dashed line indicates stitching between individual strips of eight tubes.

Additional file 3: Fig. S3. DMSO concentration optimisation: four different concentrations were used to reduce non-specific amplification on samples of *Trichobilharzia* spp. and other non-target trematodes: L: 100-bp DNA ladder (NEB), R: *T. regenti*, F: *T. franki*, S: *T. szidati*, 1: *Allobilharzia visceralis*, 2: *Bilharziella polonica*, 3: *Australapatemon burti*, 4: *Echinostoma revolutum*, 5: *Plagiorchis maculosus*, 6: *Hypoderaeum conoideum*, 7: *Schistosoma mansoni*, NTC: no template control.

## Data Availability

No datasets were generated or analysed during the current study.

## Abbreviations

CD: Cercarial dermatitis
eDNA: Environmental DNA
LAMP: Loop-mediated isothermal amplification
LoD: Limit of detection
PCR: Polymerase chain reaction
qPCR: Quantitative polymerase chain reaction

## References

[1] Horák P, Mikeš L, Lichtenbergová L, Skála V, Soldánová M, Brant SV. Avian schistosomes and outbreaks of cercarial dermatitis. Clin Microbiol Rev. 2015;28:165–90. 25567226 10.1128/CMR.00043-14 PMC4284296

[2] Macháček T, Turjanicová L, Bulantová J, Hrdý J, Horák P, Mikeš L. Cercarial dermatitis: a systematic follow-up study of human cases with implications for diagnostics. Parasitol Res. 2018;117:3881–95. 30302587 10.1007/s00436-018-6095-0

[3] Langenberg MCC, Hoogerwerf M-A, Koopman JPR, Janse JJ, Kos-van Oosterhoud J, Feijt C, et al. A controlled human *Schistosoma mansoni* infection model to advance novel drugs, vaccines and diagnostics. Nat Med. 2020;26:326–32. 32066978 10.1038/s41591-020-0759-x

[4] Horák P, Kolářová L, Adema CM. Biology of the schistosome genus *Trichobilharzia*. Adv Parasitol. 2002;52:155–233. 12521261 10.1016/s0065-308x(02)52012-1

[5] Kouřilová P, Hogg KG, Kolářová L, Mountford AP. Cercarial dermatitis caused by bird schistosomes comprises both immediate and late phase cutaneous hypersensitivity reactions. J Immunol. 2004;172:3766–74. 15004181 10.4049/jimmunol.172.6.3766

[6] Kolářová L, Horák P, Skírnisson K, Marečková H, Doenhoff M. Cercarial dermatitis, a neglected allergic disease. Clin Rev Allergy Immunol. 2013;45:63–74. 22915284 10.1007/s12016-012-8334-y

[7] Chamot E, Toscani L, Rougemont A. Public health importance and risk factors for cercarial dermatitis associated with swimming in Lake Leman at Geneva. Switzerland Epidemiol Infect. 1998;120:305–14. 9692609 10.1017/s0950268898008826 PMC2809408

[8] Schets FM, Lodder WJ, van Duynhoven YTHP, de Roda Husman AM. Cercarial dermatitis in the Netherlands caused by *Trichobilharzia* spp. J Water Health. 2008;6:187–95. 18209281 10.2166/wh.2008.028

[9] Tracz ES, Al-Jubury A, Buchmann K, Bygum A. Outbreak of swimmer’s itch in Denmark. Acta Derm Venereol. 2019;99:1116–20. 31453626 10.2340/00015555-3309

[10] Gulyás K, Soldánová M, Orosová M, Oros M. Confirmation of the presence of zoonotic *Trichobilharzia franki* following a human cercarial dermatitis outbreak in recreational water in Slovakia. Parasitol Res. 2020;119:2531–7. 32562067 10.1007/s00436-020-06751-y

[11] Caron Y, Cabaraux A, Marechal F, Losson B. Swimmer’s itch in Belgium: first recorded outbreaks, molecular identification of the parasite species and intermediate hosts. Vector-Borne Zoonotic Dis. 2017;17:190–4. 28112601 10.1089/vbz.2016.2034

[12] De Liberato C, Berrilli F, Bossù T, Magliano A, Montalbano Di Filippo M, Di Cave D, et al. Outbreak of swimmer’s itch in Central Italy: description, causative agent and preventive measures. Zoonoses Public Health. 2019;66:377–81. 30784198 10.1111/zph.12570

[13] Korycińska J, Rybak-d’obyrn J, Kubiak D, Kubiak K, Dzika E. Dermatological and molecular evidence of human cercarial dermatitis in north-eastern Poland. Vector Borne Zoonotic Dis Larchmt N. 2021;21:269–74. 10.1089/vbz.2020.2681 33566721

[14] Kerr O, Juhász A, Jones S, Stothard JR. Human cercarial dermatitis (HCD) in the UK: an overlooked and under-reported nuisance? Parasit Vectors. 2024;17:83. 38388442 10.1186/s13071-024-06176-x PMC10885386

[15] Bispo MT, Calado M, Maurício IL, Ferreira PM, Belo S. Zoonotic threats: the (re)emergence of cercarial dermatitis, its dynamics, and impact in Europe. Pathogens. 2024;13:282. 38668237 10.3390/pathogens13040282 PMC11053805

[16] Brant SV, Loker ES. Schistosomes in the southwest United States and their potential for causing cercarial dermatitis or ‘swimmer’s itch.’ J Helminthol. 2009;83:191–8. 19366484 10.1017/S0022149X09308020 PMC2892308

[17] Gordy MA, Cobb TP, Hanington PC. Swimmer’s itch in Canada: a look at the past and a survey of the present to plan for the future. Environ Health. 2018;17:73. 30359259 10.1186/s12940-018-0417-7 PMC6203143

[18] Gohardehi S, Fakhar M, Madjidaei M. Avian schistosomes and human cercarial dermatitis in a wildlife refuge in Mazandaran Province, northern Iran. Zoonoses Public Health. 2013;60:442–7. 23121919 10.1111/zph.12020

[19] Lawton SP, Lim RM, Dukes JP, Cook RT, Walker AJ, Kirk RS. Identification of a major causative agent of human cercarial dermatitis, *Trichobilharzia franki* (Müller and Kimmig 1994), in southern England and its evolutionary relationships with other European populations. Parasit Vectors. 2014;7:277. 24946974 10.1186/1756-3305-7-277 PMC4074431

[20] Kolářová L, Horák P, Skírnisson K. Methodical approaches in the identification of areas with a potential risk of infection by bird schistosomes causing cercarial dermatitis. J Helminthol. 2010;84:327–35. 20102677 10.1017/S0022149X09990721

[21] Rudko SP, Reimink RL, Froelich K, Gordy MA, Blankespoor CL, Hanington PC. Use of qPCR-based cercariometry to assess swimmer’s itch in recreational lakes. EcoHealth. 2018;15:827–39. 30120669 10.1007/s10393-018-1362-1 PMC6267424

[22] Schets FM, Lodder WJ, de Roda Husman AM. Confirmation of the presence of *Trichobilharzia* by examination of water samples and snails following reports of cases of cercarial dermatitis. Parasitology. 2010;137:77–83. 19691864 10.1017/S0031182009990849

[23] Helmer N, Hörweg C, Sattmann H, Reier S, Szucsich NU, Bulantová J, et al. DNA Barcoding of *Trichobilharzia* (Trematoda: Schistosomatidae) species and their detection in eDNA water samples. Diversity. 2023;15:104.

[24] Mahittikorn A, Thammasonthijarern N, Roobthaisong A, Udonsom R, Popruk S, Siri S, et al. Development of a loop-mediated isothermal amplification technique and comparison with quantitative real-time PCR for the rapid visual detection of canine neosporosis. Parasit Vectors. 2017;10:394. 28835287 10.1186/s13071-017-2330-2 PMC5569544

[25] Fernández-Soto P, Fernández-Medina C, Cruz-Fernández S, Crego-Vicente B, Febrer-Sendra B, García-Bernalt Diego J, et al. Whip-LAMP: a novel LAMP assay for the detection of *Trichuris muris*-derived DNA in stool and urine samples in a murine experimental infection model. Parasit Vectors. 2020;13:552. 33160406 10.1186/s13071-020-04435-1 PMC7648965

[26] Fernández-Soto P, Arahuetes JG, Hernández AS, Abán JL, Santiago BV, Muro A. A loop-mediated isothermal amplification (LAMP) assay for early detection of *Schistosoma mansoni* in stool samples: a diagnostic approach in a murine model. PLoS Negl Trop Dis. 2014;8:e3126. 25187956 10.1371/journal.pntd.0003126 PMC4154662

[27] Blin M, Senghor B, Boissier J, Mulero S, Rey O, Portela J. Development of environmental loop-mediated isothermal amplification (eLAMP) diagnostic tool for *Bulinus truncatus* field detection. Parasit Vectors. 2023;16:78. 36855192 10.1186/s13071-023-05705-4 PMC9972309

[28] Vondráček O, Mikeš L, Talacko P, Leontovyč R, Bulantová J, Horák P. Differential proteomic analysis of laser-microdissected penetration glands of avian schistosome cercariae with a focus on proteins involved in host invasion. Int J Parasitol. 2022;52:343–58. 35218763 10.1016/j.ijpara.2021.12.003

[29] Macháček T, Leontovyč R, Šmídová B, Majer M, Vondráček O, Vojtěchová I, et al. Mechanisms of the host immune response and helminth-induced pathology during *Trichobilharzia regenti* (Schistosomatidae) neuroinvasion in mice. PLoS Pathog. 2022;18:e1010302. 35120185 10.1371/journal.ppat.1010302 PMC8849443

[30] Peterková K, Konečný L, Macháček T, Jedličková L, Winkelmann F, Sombetzki M, et al. Winners vs losers: *Schistosoma**mansoni* intestinal and liver eggs exhibit striking differences in gene expression and immunogenicity. PLoS Pathog. 2024;20:e1012268. 38814989 10.1371/journal.ppat.1012268 PMC11166329

[31] Dvořák J, Vaňáčová Š, Hampl V, Flegr J, Horák P. Comparison of European *Trichobilharzia* species based on ITS1 and ITS2 sequences. Parasitology. 2002;124:307–13. 11922432 10.1017/s0031182001001238

[32] Gabrielli AF, Garba DA. Schistosomiasis in Europe. Curr Trop Med Rep. 2023;10:79–87.

[33] Kane RA, Stothard JR, Rollinson D, Leclipteux T, Evraerts J, Standley CJ, et al. Detection and quantification of schistosome DNA in freshwater snails using either fluorescent probes in real-time PCR or oligochromatographic dipstick assays targeting the ribosomal intergenic spacer. Acta Trop. 2013;128:241–9. 22100540 10.1016/j.actatropica.2011.10.019

[34] Rudko SP, Turnbull A, Reimink RL, Froelich K, Hanington PC. Species-specific qPCR assays allow for high-resolution population assessment of four species avian schistosome that cause swimmer’s itch in recreational lakes. Int J Parasitol Parasites Wildl. 2019;9:122–9. 31061794 10.1016/j.ijppaw.2019.04.006 PMC6488534

[35] Jothikumar N, Mull BJ, Brant SV, Loker ES, Collinson J, Secor WE, et al. Real-Time PCR and sequencing assays for rapid detection and identification of avian schistosomes in environmental samples. Appl Environ Microbiol. 2015;81:4207–15. 25862226 10.1128/AEM.00750-15 PMC4524150

[36] Fernández-Soto P, Gandasegui J, Rodríguez CC, Pérez-Arellano JL, Crego-Vicente B, Diego JGB, et al. Detection of *Schistosoma mansoni*-derived DNA in human urine samples by loop-mediated isothermal amplification (LAMP). PLoS ONE. 2019;14:e0214125. 30913249 10.1371/journal.pone.0214125 PMC6435178

[37] Besuschio SA, Murcia ML, Benatar AF, Monnerat S, Cruz I, Picado A, et al. Analytical sensitivity and specificity of a loop-mediated isothermal amplification (LAMP) kit prototype for detection of *Trypanosoma cruzi* DNA in human blood samples. PLoS Negl Trop Dis. 2017;11:e0005779. 28727723 10.1371/journal.pntd.0005779 PMC5544240

[38] Davis CN, Tyson F, Cutress D, Davies E, Jones DL, Brophy PM, et al. Rapid detection of *Galba truncatula* in water sources on pasture-land using loop-mediated isothermal amplification for control of trematode infections. Parasit Vectors. 2020;13:496. 32998778 10.1186/s13071-020-04371-0 PMC7526160

[39] Wang D-G, Brewster JD, Paul M, Tomasula PM. Two methods for increased specificity and sensitivity in loop-mediated isothermal amplification. Molecules. 2015;20:6048–59. 25853320 10.3390/molecules20046048 PMC6272222

[40] Takahashi M, Saccò M, Kestel JH, Nester G, Campbell MA, van der Heyde M, et al. Aquatic environmental DNA: A review of the macro-organismal biomonitoring revolution. Sci Total Environ. 2023;873:162322. 36801404 10.1016/j.scitotenv.2023.162322

[41] Podhorský M, Hůzová Z, Mikeš L, Horák P. Cercarial dimensions and surface structures as a tool for species determination of *Trichobilharzia* spp. Acta Parasitol. 2009;54:28–36.

[42] Olson PD, Cribb TH, Tkach VV, Bray RA, Littlewood DTJ. Phylogeny and classification of the Digenea (Platyhelminthes: Trematoda)1. Int J Parasitol. 2003;33:733–55. 12814653 10.1016/s0020-7519(03)00049-3

[43] Pennance T, Archer J, Lugli EB, Rostron P, Llanwarne F, Ali SM, et al. Development of a molecular snail xenomonitoring assay to detect *Schistosoma haematobium* and *Schistosoma bovis* infections in their *Bulinus* snail hosts. Molecules. 2020;25:4011. 32887445 10.3390/molecules25174011 PMC7116084

[44] Schols R, Carolus H, Hammoud C, Mulero S, Mudavanhu A, Huyse T. A rapid diagnostic multiplex PCR approach for xenomonitoring of human and animal schistosomiasis in a “One Health” context. Trans R Soc Trop Med Hyg. 2019;113:722–9. 31369105 10.1093/trstmh/trz067

[45] Pennance T, Lam Y, Bigot N, Trapp J, Spaan JM, Ogara G, et al. A rapid diagnostic PCR assay for the detection of *Schistosoma mansoni* in their snail vectors. J Parasitol. 2024;110:684–9. 39701157 10.1645/24-44 PMC12175291

[46] Blin M, Dametto S, Agniwo P, Webster BL, Angora E, Dabo A, et al. A duplex tetra-primer ARMS-PCR assay to discriminate three species of the *Schistosoma haematobium* group: *Schistosoma**curassoni*, *S.**bovis*, *S.**haematobium* and their hybrids. Parasit Vectors. 2023;16:121. 37029440 10.1186/s13071-023-05754-9 PMC10082484

