# Supplementary figures and images for: Molecular detection and identification of Trichobilharzia: development of a LAMP, qPCR, and multiplex PCR toolkit
Source: Parasit Vectors. 2025 May 30;18:195. doi: 10.1186/s13071-025-06822-y (PMC12124058; doi:10.1186/s13071-025-06822-y)

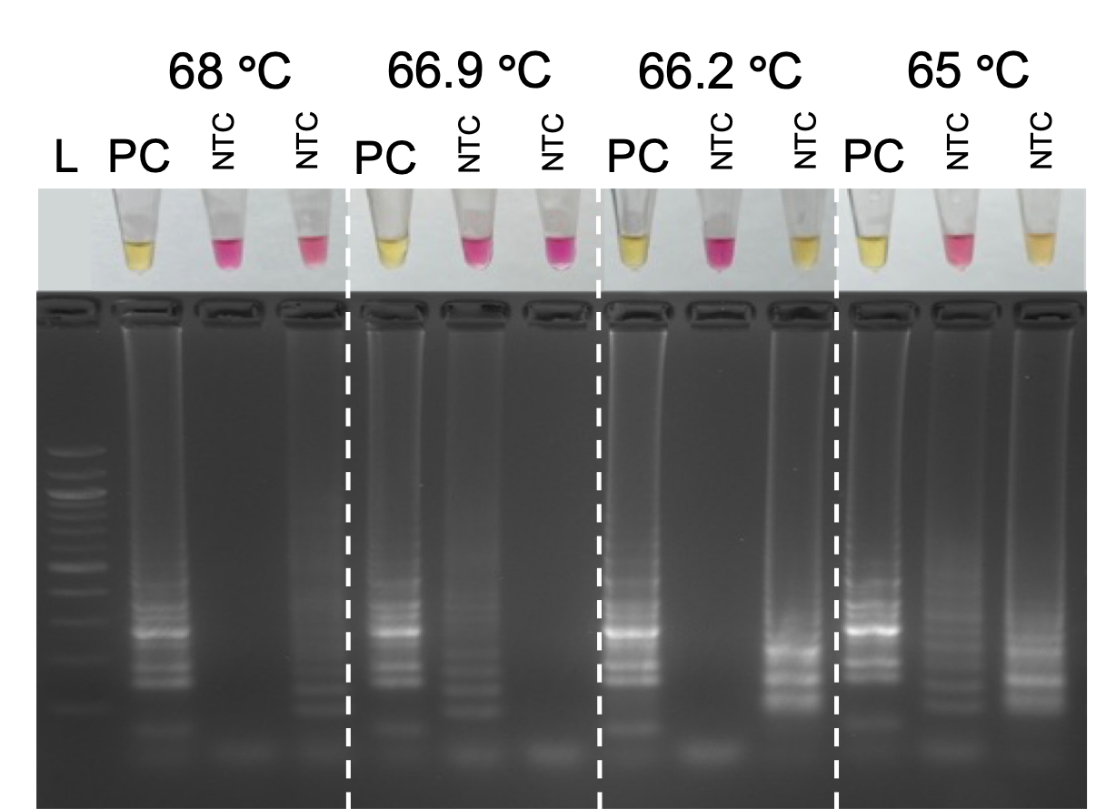

Supplement: Supplementary file 1 — Additional file 1: Fig. S1. Temperature gradient used to select the optimal temperature for reduction of false-positives. Tested temperatures are shown above individual sections separated by white dashed lines. L: 100-bp DNA ladder, PC: positive control (1 ng Trichobilharzia gDNA), NTC: no template control in duplicate. [file 13071_2025_6822_MOESM1_ESM.tiff]

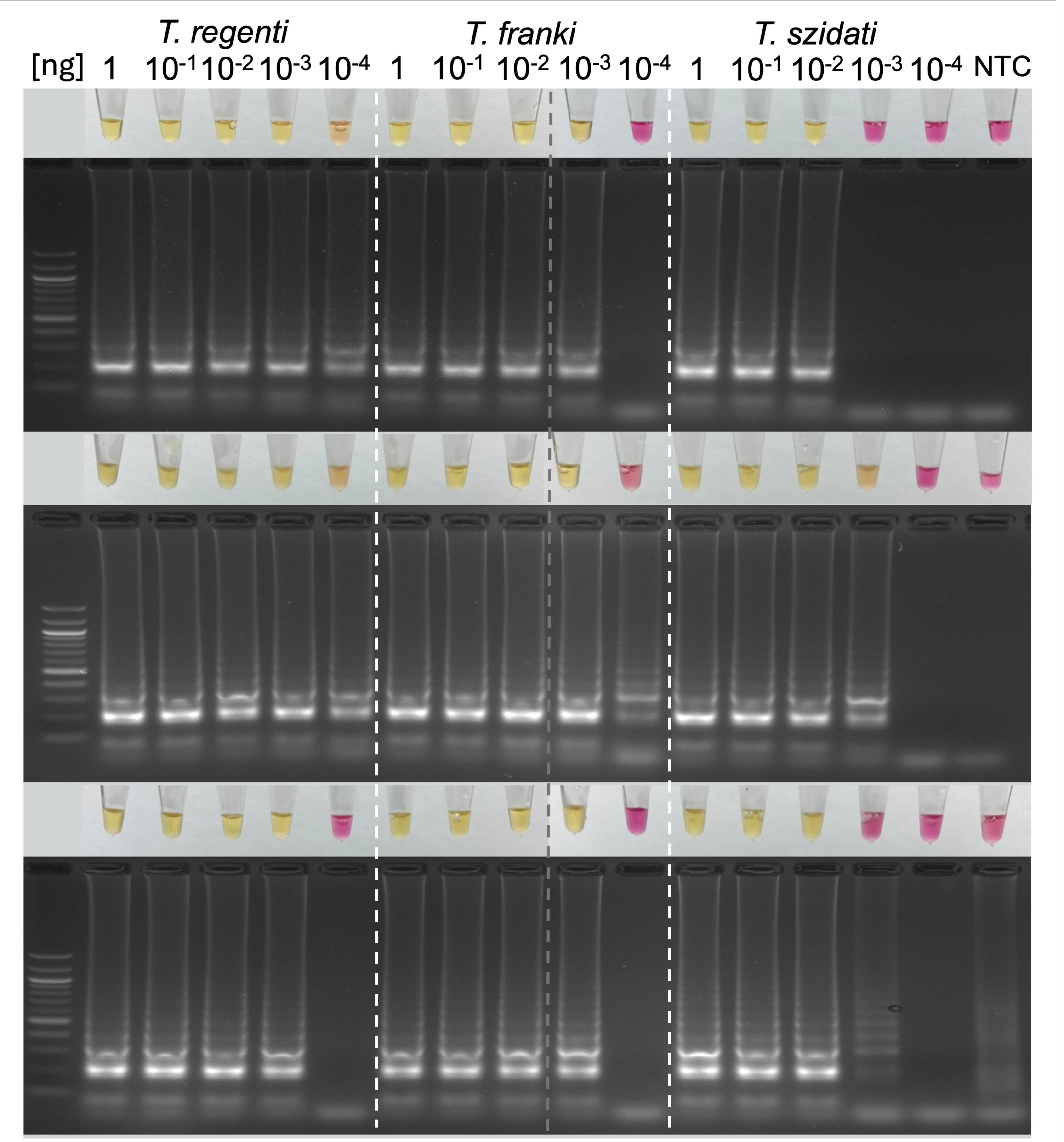

Supplement: Supplementary file 2 — Additional file 2: Fig. S2. Specificity and sensitivity testing on dilution series (1–10−4 ng) of gDNA of three species (Trichobilharzia regenti, T. franki, and T. szidati). Each row corresponds to one replicate. NTC: no template control. Grey dashed line indicates stitching between individual strips of eight tubes. [file 13071_2025_6822_MOESM2_ESM.tiff]

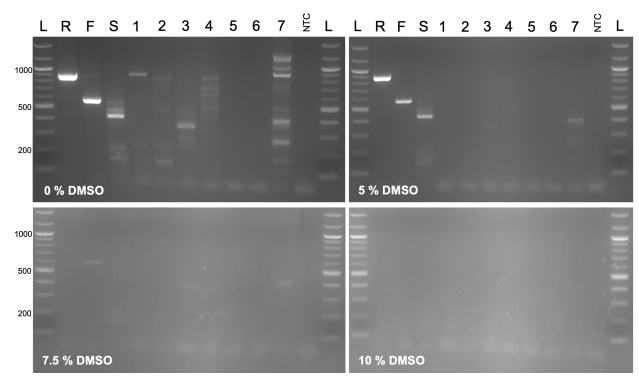

Supplement: Supplementary file 3 — Additional file 3: Fig. S3. DMSO concentration optimisation: four different concentrations were used to reduce non-specific amplification on samples of Trichobilharzia spp. and other non-target trematodes: L: 100-bp DNA ladder (NEB), R: T. regenti, F: T. franki, S: T. szidati, 1: Allobilharzia visceralis, 2: Bilharziella polonica, 3: Australapatemon burti, 4: Echinostoma revolutum, 5: Plagiorchis maculosus, 6: Hypoderaeum conoideum, 7: Schistosoma mansoni, NTC: no template control. [file 13071_2025_6822_MOESM3_ESM.tiff]
